# Cardiovascular safety of tocilizumab: A systematic review and network meta-analysis

**DOI:** 10.1371/journal.pone.0220178

**Published:** 2019-08-01

**Authors:** Benjamin Castagné, Marie Viprey, Julie Martin, Anne-Marie Schott, Michel Cucherat, Martin Soubrier

**Affiliations:** 1 Rheumatology Department, Gabriel-Montpied University Hospital, Clermont-Ferrand, France; 2 HESPER EA 7425, University of Lyon, Claude Bernard University Lyon 1, Lyon, France; 3 Public Health Centre, Hospices Civils de Lyon, Lyon, France; 4 University Lyon, UMR 5558, Laboratory of Biometry and Evolutionary Biology, CNRS, Villeurbanne, France; 5 Department of Pharmacology and Toxicology, Hospices Civils de Lyon, Lyon, France; University of Mississippi Medical Center, UNITED STATES

## Abstract

**Objectives:**

Our objective was to compare the cardiovascular safety of tocilizumab and other biological disease-modifying antirheumatic drugs (bDMARD) in rheumatoid arthritis using a network meta-analysis (NMA).

**Methods:**

A systematic literature search through May 2018 identified randomized controlled trials (RCT) or observational studies (cohort only) reporting cardiovascular outcomes of tocilizumab (TCZ) and/or abatacept (ABA) and/or rituximab (RTX) and/or tumor necrosis factor inhibitors (TNFi) in rheumatoid arthritis patients. The composite primary outcome was the rate of major adverse cardiovascular outcomes (MACE, myocardial infarction (MI), peripheral artery disease (PAD) and cardiac heart failure (CHF)).

**Results:**

19 studies were included in the NMA, including 11 RCTs and 8 cohort studies. We found less events with RTX (5.41 [1.70;17.26]. We found no difference between TCZ and other treatments. Concerning MI, we found no difference between TCZ and csDMARD (4.23 [0.22;80.64]), no difference between TCZ and TNFi (2.00 [0.18;21.84]). There was no difference between TCZ and csDMARD (1.51[0.02;103.50] and between TCZ and TNFi (1.00 [0.06;15.85]) for stroke event.

With cohorts and RCT NMA, we found no difference between TCZ and other treatments for MACE (0.66 [0.42;1.03] with ABA, 1.04 [0.60;1.81] with RTX, 0.78[0.53;1.16] and 0.91 [0.54;1.51] with csDMARD), but the risk of myocardial infarction was lower with TCZ compared to ABA (0.67 [0.47;0.97]).

We lacked data to compare TCZ and other bDMARD for stoke and MI. Not enough data was available to perform a NMA for CHF and PAD.

**Conclusions:**

Despite an increase in cholesterol levels, TCZ has safe cardiovascular outcomes compared to other bDMARD.

## Introduction

Rheumatoid Arthritis (RA) is one of the most frequent chronic inflammatory rheumatism (CIR), characterized by chronic inflammation of joints, particularly hands and feet. The management of RA has been revolutionized with the development of biological disease-modifying antirheumatic drugs (bDMARD) [1,2]. Mortality is increased in RA, especially due to cardiovascular diseases (CVD) [3,4]. In a Danish population based study, the risk of CVD was similar between RA and diabetes (RR 1.7 95% CI 1.5 to 6.9 vs RR 1.7 95% CI 1.6 to 1.8, respectively, p = 0.64) [5]. The cardiovascular risk seems to be linked with the disease’s activity. In a recent study, Arts & al showed that low disease activity (DAS28 ≤3.2) was associated with a reduced risk of CVD (HR 0.65 95% CI 0.43 to 0.99) compared with moderate or high disease activity (DAS28 >3.2) [6].

The release of inflammatory cytokines (IL6, IL1, TNF alpha) leads to a decrease of total cholesterol (CT), especially HDL-cholesterol (HDL-C) [7,8]. Furthermore, the anti-atherogenic function of HDL-cholesterol become pro-atherogenic because of a linkage with inflammatory proteins like SAA [8]. In the recent recommendations of the European Ligue Against Rheumatism (EULAR), CVD risk assessment is recommended for all patients with RA at least once every 5 years [9].

Tocilizumab (TCZ) is a bDMARD which blockades the IL-6 receptor and has demonstrated its efficacy to control the disease activity in a phase III double-blinded randomized controlled trial (RCT): the OPTION study [10]. However, RCT and other studies showed that TCZ was associated with an increased cholesterol level [10,11]. This increase was also estimated superior compared to adalimumab (ADA) in a post-hoc analysis of the ADACTA trial [12]. TCZ interacts with CYP450 which is also implicated in atorvastatin metabolism [13]. Therefore, it has been used with caution in patients with high cardiovascular risk, particularly those with dyslipidemia. Recent studies showed reassuring data concerning major adverse cardiovascular events (MACE) with TCZ compared to other bDMARD [14–18]. Two studies conducted in American databases underscored significant decrease in cardiovascular events with TCZ [14,15]. In Zhang & al. study, TCZ reduced the risk of myocardial infarction (MI) significantly more than abatacept (ABA) (HR 0.64 95% CI 0.41 to 0.99) [14]. Kim & al. found better cardiovascular outcomes with TCZ comparing to TNF inhibitors (TNFi) (HR 0.68 95% CI 0.49 to 0.94) [15]. Furthermore, TCZ decreases inflammatory proteins like SAA, and may restore the anti-atherogenic function of HDL-C.

Given these controversial results, we aimed to assess the cardiovascular safety of tocilizumab in rheumatoid arthritis compared to other bDMARD using a network meta-analysis (NMA).

## Materials and methods

This systematic review with meta-analysis was conducted and reported according to the Preferred Reporting Items for Systematic Reviews and Meta-Analysis statement (PRISMA) guidelines (S1 File) [19]. Our protocol was recorded on PROSPERO under the registration number CRD42018097180.

### Literature search

This systematic review was performed by searching in PubMed/Medline, Science Direct, and Web of Science databases, and in Cochrane and Wiley Online libraries. We also searched abstracts in the American College of Rheumatology (ACR) and European Ligue Against Rheumatism (EULAR) annual meetings databases, from January 2003 until May 2018. The search equations are available in supporting information (S2 File).

### Eligibility criteria

Our eligibility criteria for qualitative analysis were: RCT or observational (cohort only) studies, wrote in French or in English, assessing cardiovascular outcomes among rheumatoid arthritis patients treated by TCZ and/or ABA and/or rituximab (RTX) and/or TNFi.

### Study selection

Studies were selected on the basis of their titles and abstracts, then on their full text, by two independent reviewers (BC, JM). Disagreements were resolved through discussion with a third reviewer (MV), when necessary.

### Data extraction and quality assessment

Two reviewers (BC and JM) assessed independently the quality of selected studies using the Cochrane Risk Of Bias tool (RoB 2.0) [20] for randomized studies and the Cochrane Risk Of Bias In Non-randomized Studies—of Interventions (ROBINS-I tool) [21] for non-randomized. The two reviewers (BC and JM) also performed data extraction independently using a predetermined form. If necessary, disagreements were resolved by the third reviewer (MV). We extracted from each study: authors and publication year, country, bDMARD used in the study, number of previous bDMARD and previous csDMARD, number of patients, patient-year, follow-up period (year), number of MACE (major adverse cardiovascular events), myocardial infarction (MI), stroke, cardiac heart failure (CHF) and peripheral artery disease (PAD),

### Outcomes measures

The composite primary outcome was the rate of MACE which included stroke, MI, CHF and PAD (obliterating arteriopathy of the limbs, kidney and mesenteric artery diseases and aortic diseases. The secondary outcomes were rates of each cardiovascular event).

### Data analysis

A frequentist network meta-analysis based on a random effects model [22] was conducted for all outcomes to compute relative risk (RR) and their 95% confidence interval (95%CI). NMA allows to synthesize information from numerous studies addressing the same outcomes but involving different interventions [23,24]. NMA combines direct and indirect evidence across a network of studies into a single effect size. Forest plots were used to represent the quantitative results. For pairwise comparison, statistical heterogeneity between studies was assessed by the Cochran’s Q test (p<0.05 for significativity) and the I^2^ statistic (<25%: low heterogeneity, 25–50%: moderate, 50–75%: high and >75%: very high). All analyses were performed using R (netmeta package version 0.9–5 for treatment comparison, meta package version 4.8–2, R Language and Environment for Statistical Computing, Vienna, Austria) [25,26]. A subgroup analysis was performed, including RCTs only and cohort studies only.

## Results

### Literature search results and studies characteristics

**Fig 1:**
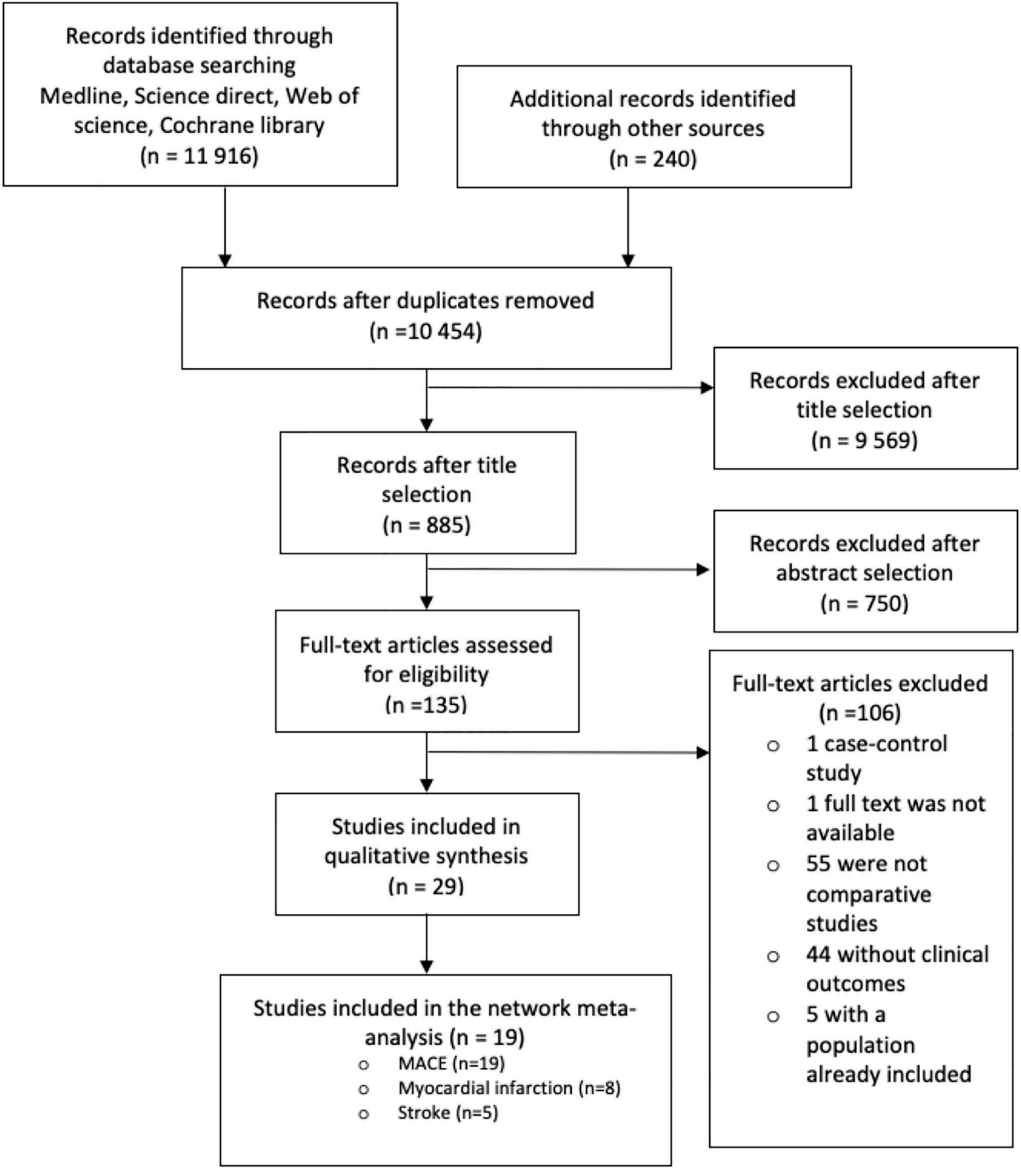
Flow chart of the systematic literature review. PubMed/Medline, Web of science, Cochrane library, Science direct and ACR, Annual College of Rheumatology; EULAR, European League Against Rheumatism databases.

After duplicates removal, 10 454 articles were found using PubMed/Medline, Science direct, Web of science, Cochrane library databases and ACR and EULAR conference abstracts. Of the 10 454 identified records, 10 319 were excluded at title/abstract level, leaving 135 full-text examined. Of these, we selected 29 studies, 27 original articles and two conference abstracts [18,27] for qualitative analysis (Fig 1). 106 articles did not meet the inclusion criteria for the following reasons: one was a case-control study, 55 were not controlled studies, 44 did not have clinical outcomes (biological or imaging), five because the studied population was already included, and one because the full-test could not be accessed.

Of the 29 studies included in the qualitative analysis, 13 studies were conducted in North America [11,14,15,17,28–35], 16 in Europe [10,11,17,18,29,34,36–45], five in Asia [17,32,46–48], four in South America [11,17,34,38] and two in Australia [11,32]. Study characteristics are available in Table 1. 16/29 studies were RCT [10,11,27,29,32,34–36,39,41–46,48]and 13/29 were cohort studies [14,15,17,18,28,30,31,33,37,38,40,47,49]. Of the 13 cohort studies, five [14,15,17,31,33] were performed using national health care databases and four [14,15,31,33] were propensity matched. Of the 16 RCT, four were open-label trials [27,41,43,46], the others were double-blinded.

**Table 1:** Characteristics and results of studies on cardiovascular events (n = 29).

STUDY	TREATMENT	AGE (YEARS)	GENDER (FEMALE)	HTA	DYSLIPIDEMIA	SMOKING	DIABETUS MIELLETUS	PREV CV	PREVB	PREVCS	N	TOTAL PY	FOLLOW-UP (YEAR)	MACE	MI	STROKE	CHF
**AL-ALY &AL.** [28] **2011**	TNFi	57 ±12	9%	68%	52%	NS	31%	38%	NS	NS	3796	9 563	3.5	1460	NS	NS	NS
	csDMARD	63 ±12	9%	68%	52%	NS	31%	36%	NS	NS	19899	65 766	3.5	7761	NS	NS	NS
**BURMESTER &AL.** [36] **2017**	TCZ	50.2±13.6	77%	NS	NS	NS	NS	NS	0	0	1 108	1 645	2	NS	NS	NS	NS
	MTX	49.6 ±13.1	80%	NS	NS	NS	NS	NS	0	0	282	339	2	NS	NS	NS	NS
**CARDENAS &AL.** [37] **2016**	IFX	51.5 ±12.7	80%	NS	NS	NS	NS	NS	0	1	55	NS	2	3	NS	NS	NS
	ADA	52.9 ±14.7	87%	NS	NS	NS	NS	NS	0	1	31	NS	2	1	NS	NS	NS
	ETN	55.1 ±14	82%	NS	NS	NS	NS	NS	0	1	44	NS	2	3	NS	NS	NS
**CHOY &AL.** [38] **2017**	TCZ	54.3 ±12.8	NS	NS	NS	NS	NS	NS	0	1	423	404	1	0	NS	0	NS
	TNFi	55.2 ±13.1	NS	NS	NS	NS	NS	NS	0	1	793	776	1	2	NS	2	NS
**CURTIS &AL.** [17] **2015**	TCZ (insurance claims databases)	NS	NS	NS	NS	NS	NS	NS	NS	NS	62 713	60 754	NS	206	115	91	NS
	TNFi	NS	NS	NS	NS	NS	NS	NS	NS	NS	19 000	53 360	NS	676	308	368	NS
	TCZ (safety database)	NS	NS	NS	NS	NS	NS	NS	NS	NS	5 734	4 345	NS	28	14	14	NS
**EMERY &AL.** [29] **2010**	CTZ	50.4 ±13.6	76%	NS	NS	NS	NS	NS	0	0	659	NS	1	39	NS	NS	NS
	MTX	51.2 ±13	80%	NS	NS	NS	NS	NS	0	0	213	NS	1	9	NS	NS	NS
**EMERY &AL.** [39] **2017**	RTX	51.6 ±12.7	80.4%	NS	NS	NS	NS	NS	0	1	337	161	0.5	4	NS	NS	NS
	MTX	52.16±12.4	85.5%	NS	NS	NS	NS	NS	0	1	172	79	0.5	13	NS	NS	NS
**GABAY &AL.** [11] **2013**	TCZ	54.4 ±13	79%	NS	NS	NS	NS	NS	0	1	162	NS	0.5	3	2	1	NS
	ADA	53.3 ±12.4	82%	NS	NS	NS	NS	NS	0	1	162	NS	0.5	2	1	1	NS
**GEBOREK&AL.** [49] **2002**	ETN	54	78%	NS	NS	NS	NS	NS	0	2	166	NS	NS	4	4	0	0
	IFX	55.4	79%	NS	NS	NS	NS	NS	0	2	135	NS	NS	0	0	0	0
	csDMARD	61.3	82%	NS	NS	NS	NS	NS	0	2	103	NS	NS	0	0	0	0
**GILES &AL.** [27] **2016**	TCZ	61	78%	71%	NS	29%	18%	NS	0	1	1538	4900	3.2	83	29	26	12
	ETN								0	12	1542	4891	3.2	78	32	16	8
**GOTTENBERG &AL.** [18] **2016**	TCZ	NS	NS	NS	NS	NS	NS	NS	NS	NS	NS	3 441	2	NS	NS	NS	NS
	ABA	NS	NS	NS	NS	NS	NS	NS	NS	NS	NS	4 912	2	NS	NS	NS	NS
	RTX	NS	NS	NS	NS	NS	NS	NS	NS	NS	NS	10 545	2	NS	NS	NS	NS
**HARROLD &AL.** [30] **2015**	RTX	57.8 ±11.7	81%	NS	NS	NS	9.8%	11.3%	≧1	NS	265	242	1	5	NS	NS	NS
	TNFi	56.1 ±12.4	79%	NS	NS	NS	10.7%	7.3%	≧1	NS	205	737	1	9	NS	NS	NS
**IANNONE &AL.** [40] **2017**	TCZ	54.5		14.3%	NS	NS	3.9%	3.5%	NS	NS	202	NS	2	7	NS	NS	NS
	ABA	57.4	77.2%	15.3%	NS	NS	2.9%	4.7%	NS	NS	230	NS	2	11	NS	NS	NS
	TNFi	53,9		23.2%	NS	NS	4.9%	5%	NS	NS	1 135	NS	2	33	NS	NS	NS
**JIN &AL.** [31] **2017**	ABA	NS	NS	NS	NS	NS	NS	NS	NS	NS	6934	NS	NS	114	NS	NS	NS
	TNFi	NS	NS	NS	NS	NS	NS	NS	NS	NS	6934	NS	NS	113	NS	NS	NS
**JOBANPUTRA &AL.** [41] **2012**	ADA	55 ±12.5	75%	NS	NS	NS	NS	NS	0	2	60	NS	1	5	NS	NS	NS
	ETN	53.2 ±12.4	70%	NS	NS	NS	NS	NS	0	2	60	NS	1	6	NS	NS	NS
**KAY &AL.** [42] **2008**	GOL	54 (46–64)	77.4%	NS	NS	NS	NS	NS	0	1	137	NS	1	2	NS	NS	2
	MTX	52 (46–66)	74.3%	NS	NS	NS	NS	NS	0	1	35	NS	1	0	NS	NS	0
**KEYSTONE &AL.** [32] **2016**	GOL	51 (44–57)	89%	NS	NS	NS	NS	NS	0	1	434	NS	5	2	2	NS	NS
	MTX	52 (42–58)	82%	NS	NS	NS	NS	NS	0	1	105	NS	5	0	0	NS	NS
**KIM &AL.** [46] **2012**	ETN	48.4 ±12	91.4%	NS	NS	NS	NS	NS	0	0	137	NS	0.33	1	NS	NS	1
	csDMARD	48.5 ±11.3	88.4%	NS	NS	NS	NS	NS	0	0	103	NS	0.33	0	NS	NS	0
**KIM &AL.** [15] **2017**	TCZ	58.9 ±10,2	88,3%	60,6%	45,8%	NS	20%	12,5%	≧1	NS	9 218	7 236	NS	43	21	23	NS
	TNFi	58.7 ±10	82%	57,5%	44%	NS	16%	12.2%	≧1	NS	18 810	14 776	NS	103	7	49	NS
**KIM &AL.** [33] **2018**	TCZ	58,8 ±10,4	81,4%	59,8%	46.3%	NS	20,1%	12%	NS	NS	6 237	N S	NS	32	NS	NS	NS
	ABA	59 ±10	87%	59,1%	45.4%	NS	19,23%	12,6	NS	NS	14 685	NS	NS	112	NS	NS	NS
**MANDERS &AL.** [43] **2015**	ABA	56.2 ±9.95	88.4%	NS	NS	NS	NS	NS	1	1	43	NS	1	1	NS	NS	NS
	RTX	57.1 ±11.1	63%	NS	NS	NS	NS	NS	1	1	46	NS	1	2	NS	NS	NS
	TNFi	56.3 ±11.2	74%	NS	NS	NS	NS	NS	1	1	50	NS	1	2	NS	NS	NS
**SAKAI & AL.** [47] **2015**	TCZ	59.2 ±13	82.5%	NS	NS	NS	10.9%	NS	NS	NS	302	224,68	1	NS	NS	NS	NS
	TNFi	57.33 ±15.2	82.8%	NS	NS	NS	10.5%	NS	NS	NS	304	231,01	1	NS	NS	NS	NS
**SMOLEN &AL.** [44] **2015**	CTZ	53.6 ±11.9	86.4%	NS	NS	NS	NS	NS	0	1	96	NS	2	9	NS	NS	NS
	csDMARD	54 ±12.4	76.4%	NS	NS	NS	NS	NS	0	1	98	NS	2	5	NS	NS	NS
**SMOLEN &AL.** [45] **2016**	CTZ	53.5 ±12.3	79%	NS	NS	NS	NS	NS	0	1	516	NS	2	12	NS	NS	9
	ADA	52.9 ±12.8	79%	NS	NS	NS	NS	NS	0	1	523	NS	2	9	NS	NS	8
**SMOLEN &AL.** [10] **2008**	TCZ	51.1 ±12.3	83.5%	NS	NS	NS	NS	NS	0	1	418	NS	1	29	NS	NS	NS
	MTX	50.6 ±12.1	78%	NS	NS	NS	NS	NS	0	1	204	NS	1	10	NS	NS	NS
**WESTHOVENS &AL.** [34] **2006**	IFX	52.5 (44_60.5)	78.9	NS	NS	NS	NS	NS	0	1	721	NS	0.5	5	4	1	NS
	MTX	52 (44–61)	83.2%	NS	NS	NS	NS	NS	0	1	363	NS	0.5	0	0	0	NS
**WEISMAN &AL.** [35] **2007**	ETN	60.6 (19–84)	72.2%	63.5%	63.5%	NS	49.7%	60%	0	1	266	NS	0.33	7	NS	NS	NS
	Placebo	59.2 (23–85)	78.1%	56.1%	66.1%	NS	49.5%	65%	0	1	269	NS	0.33	1	NS	NS	NS
**YAMAMOTO &AL.** [48] **2014**	CTZ	53.4 v±10.7	81.9%	NS	NS	NS	NS	NS	0	1	239	NS	0.5	2	2	NS	NS
	MTX	51.9 ±11.1	85.7%	NS	NS	NS	NS	NS	0	1	77	NS	0.5	0	0	NS	NS
**ZHANG &AL.** [14] **2016**	TCZ	63.7	87%	27.8%	NS	23.59%	14.1%	NS	NS	NS	3 332	2 728	NS	17	17	NS	NS
	RTX	64.9	84.4%	30%	NS	21.55%	14.84%	NS	NS	NS	7 475	8 424	NS	71	71	NS	NS
	TNFi	67.5%	86.7%	27.7%	NS	21.19%	15%	NS	NS	NS	35 718	44 763	NS	359	359	NS	NS
	ABA	65.6	87%	29.6%	NS	22.1%	13.9%	NS	NS	NS	13 608	18 747	NS	138	138	NS	NS

RTX, rituximab; ADA, adalimumab; IFX, infliximab; ETN, etanercept; CTZ, certolizumab pegol; GOL, golimumab; MTX, methotrexate; TNFi, TNF inhibitor; Prevcs, previous csDMARD, conventional synthetic disease-modifying antirheumatic drugs; PrevB, previous bDMARD biological disease-modifying antirheumatic drugs; PrevCV, previous cardiovascular event; NS, not specified; PY, patient year

We identify two major concerns due to cofounding in two cohort studies [40,49], because of the absence of multivariate analysis. The other potential bias was selective reporting due to the absence of online recorded protocol for 8 studies. Detailed bias assessment is reported in Table 2.

**Table 2:** Risk of bias assessment (n = 29).

Randomized controlled Trial (ROB2 tool)								
**Author**	**Year**	**Study acronym**	**Random sequence generation**	**Allocation concealment**	**Blinding of particpants and personnel**	**Blinding of outcome data**	**Incomplete outcome data**	**Selective reporting**
Burmester &al. [36]	2017	FUNCTION	$\begin{array}{c} + \end{array}$	$\begin{array}{c} + \end{array}$	$\begin{array}{c} + \end{array}$	$\begin{array}{c} + \end{array}$	$\begin{array}{c} + \end{array}$	$\begin{array}{c} + \end{array}$
Emery &al. * [29]	2010	SERENE	$\begin{array}{c} + \end{array}$	$\begin{array}{c} + \end{array}$	$\begin{array}{c} + \end{array}$	$\begin{array}{c} + \end{array}$	$\begin{array}{c} + \end{array}$	$\begin{array}{c} + \end{array}$
Emery &al. * [39]	2017	C-EARLY	$\begin{array}{c} + \end{array}$	$\begin{array}{c} + \end{array}$	$\begin{array}{c} + \end{array}$	$\begin{array}{c} + \end{array}$	$\begin{array}{c} + \end{array}$	$\begin{array}{c} + \end{array}$
Gabay &al. * [11]	2013	ADACTA	$\begin{array}{c} + \end{array}$	$\begin{array}{c} + \end{array}$	$\begin{array}{c} + \end{array}$	$\begin{array}{c} + \end{array}$	$\begin{array}{c} + \end{array}$	$\begin{array}{c} + \end{array}$
Giles &al. * [27]	2016	-	$\begin{array}{c} + \end{array}$	$\begin{array}{c} + \end{array}$	?	?	$\begin{array}{c} + \end{array}$	$\begin{array}{c} + \end{array}$
Jobanputra &al. [41]	2012	RED SEA	$\begin{array}{c} + \end{array}$	$\begin{array}{c} + \end{array}$	?	$\begin{array}{c} + \end{array}$	$\begin{array}{c} + \end{array}$	$\begin{array}{c} + \end{array}$
Kay &al. * [42]	2008	-	$\begin{array}{c} + \end{array}$	$\begin{array}{c} + \end{array}$	$\begin{array}{c} + \end{array}$	$\begin{array}{c} + \end{array}$	$\begin{array}{c} + \end{array}$	$\begin{array}{c} + \end{array}$
Keystone &al.* [32]	2016	GO-FORWARD	$\begin{array}{c} + \end{array}$	$\begin{array}{c} + \end{array}$	$\begin{array}{c} + \end{array}$	$\begin{array}{c} + \end{array}$	$\begin{array}{c} + \end{array}$	$\begin{array}{c} + \end{array}$
Kim &al. * [46]	2012	APPEAL	$\begin{array}{c} + \end{array}$	$\begin{array}{c} + \end{array}$	?	$\begin{array}{c} + \end{array}$	$\begin{array}{c} + \end{array}$	$\begin{array}{c} + \end{array}$
Manders &al. * [43]	2015	DREAM-TIME	$\begin{array}{c} + \end{array}$	$\begin{array}{c} + \end{array}$	?	$\begin{array}{c} + \end{array}$	$\begin{array}{c} + \end{array}$	$\begin{array}{c} + \end{array}$
Smolen &al. * [10]	2008	OPTION	$\begin{array}{c} + \end{array}$	$\begin{array}{c} + \end{array}$	$\begin{array}{c} + \end{array}$	$\begin{array}{c} + \end{array}$	$\begin{array}{c} + \end{array}$	$\begin{array}{c} + \end{array}$
Smolen &al. [45]	2016	EXXELARATE	$\begin{array}{c} + \end{array}$	$\begin{array}{c} + \end{array}$	$\begin{array}{c} + \end{array}$	$\begin{array}{c} + \end{array}$	$\begin{array}{c} + \end{array}$	$\begin{array}{c} + \end{array}$
Smolen &al. * [44]	2015	CERTAIN	$\begin{array}{c} + \end{array}$	$\begin{array}{c} + \end{array}$	$\begin{array}{c} + \end{array}$	$\begin{array}{c} + \end{array}$	$\begin{array}{c} + \end{array}$	$\begin{array}{c} + \end{array}$
Westhovens &al. * [34]	2006	-	$\begin{array}{c} + \end{array}$	$\begin{array}{c} + \end{array}$	$\begin{array}{c} + \end{array}$	$\begin{array}{c} + \end{array}$	$\begin{array}{c} + \end{array}$	$\begin{array}{c} + \end{array}$
Weisman &al. [35]	2007	-	$\begin{array}{c} + \end{array}$	$\begin{array}{c} + \end{array}$	$\begin{array}{c} + \end{array}$	$\begin{array}{c} + \end{array}$	$\begin{array}{c} + \end{array}$	$\begin{array}{c} + \end{array}$
Yamamoto &al. * [48]	2014	J-RAPID	$\begin{array}{c} + \end{array}$	$\begin{array}{c} + \end{array}$	$\begin{array}{c} + \end{array}$	$\begin{array}{c} + \end{array}$	$\begin{array}{c} + \end{array}$	$\begin{array}{c} + \end{array}$
Non randomized trials(ROBINS-I tool)								
**Author**	**Year**	**Bias due cofounding**	**Bias in selection of participatnts**	**Bias in classification of interventio**	**Bias due to deviations froms interventions**	**Bias in measurement outcome**	**Incomplete outcome data**	**Selective reporting**
Al-aly &al.* [28]	2011	****?****	$\begin{array}{c} + \end{array}$	$\begin{array}{c} + \end{array}$	$\begin{array}{c} + \end{array}$	$\begin{array}{c} + \end{array}$	$\begin{array}{c} + \end{array}$	?
Cardenas &al. [37]	2016	?	$\begin{array}{c} + \end{array}$	$\begin{array}{c} + \end{array}$	$\begin{array}{c} + \end{array}$	$\begin{array}{c} + \end{array}$	$\begin{array}{c} + \end{array}$	?
Choy &al. * [38]	2017	?	$\begin{array}{c} + \end{array}$	$\begin{array}{c} + \end{array}$	$\begin{array}{c} + \end{array}$	$\begin{array}{c} + \end{array}$	$\begin{array}{c} + \end{array}$	$\begin{array}{c} + \end{array}$
Curtis &al. [17]	2015	?	$\begin{array}{c} + \end{array}$	$\begin{array}{c} + \end{array}$	$\begin{array}{c} + \end{array}$	$\begin{array}{c} + \end{array}$	$\begin{array}{c} + \end{array}$	$\begin{array}{c} + \end{array}$
Geborek &al. * [49]	2002	****-****	$\begin{array}{c} + \end{array}$	$\begin{array}{c} + \end{array}$	$\begin{array}{c} + \end{array}$	$\begin{array}{c} + \end{array}$	$\begin{array}{c} + \end{array}$	?
Gottenberg &al. [18]	2016	?	$\begin{array}{c} + \end{array}$	$\begin{array}{c} + \end{array}$	$\begin{array}{c} + \end{array}$	$\begin{array}{c} + \end{array}$	$\begin{array}{c} + \end{array}$	?
Harrold &al. * [30]	2015	?	$\begin{array}{c} + \end{array}$	$\begin{array}{c} + \end{array}$	$\begin{array}{c} + \end{array}$	$\begin{array}{c} + \end{array}$	$\begin{array}{c} + \end{array}$	****$\begin{array}{c} + \end{array}$****
Iannone &al. * [40]	2018	****-****	$\begin{array}{c} + \end{array}$	$\begin{array}{c} + \end{array}$	$\begin{array}{c} + \end{array}$	$\begin{array}{c} + \end{array}$	$\begin{array}{c} + \end{array}$	?
Jin &al. [31]	2017	$\begin{array}{c} + \end{array}$	$\begin{array}{c} + \end{array}$	$\begin{array}{c} + \end{array}$	$\begin{array}{c} + \end{array}$	$\begin{array}{c} + \end{array}$	$\begin{array}{c} + \end{array}$	?
Kim &al. [15]	2017	$\begin{array}{c} + \end{array}$	$\begin{array}{c} + \end{array}$	$\begin{array}{c} + \end{array}$	$\begin{array}{c} + \end{array}$	$\begin{array}{c} + \end{array}$	$\begin{array}{c} + \end{array}$	$\begin{array}{c} + \end{array}$
Kim &al. * [33]	2018	$\begin{array}{c} + \end{array}$	$\begin{array}{c} + \end{array}$	$\begin{array}{c} + \end{array}$	$\begin{array}{c} + \end{array}$	$\begin{array}{c} + \end{array}$	$\begin{array}{c} + \end{array}$	$\begin{array}{c} + \end{array}$
Sakai &al. [47]	2015	****?****	$\begin{array}{c} + \end{array}$	$\begin{array}{c} + \end{array}$	$\begin{array}{c} + \end{array}$	$\begin{array}{c} + \end{array}$	$\begin{array}{c} + \end{array}$	?
Zhang &al. * [14]	2016	****$\begin{array}{c} + \end{array}$****	$\begin{array}{c} + \end{array}$	$\begin{array}{c} + \end{array}$	$\begin{array}{c} + \end{array}$	$\begin{array}{c} + \end{array}$	$\begin{array}{c} + \end{array}$	?

RTX, rituximab; ADA, adalimumab; IFX, infliximab; ETN, etanercept; CTZ, certolizumab pegol; GOL, golimumab; MTX, methotrexate; TNFi, TNF inhibitor; csDMARD, conventional synthetic disease-modifying antirheumatic drugs; NS, not specified, PY; patient year; IR incidence rate; RCT randomized controlled trial; ACR, American College of Rheumatology; EULAR, European League Against Rheumatism; + low risk; ? some concerns; − high risk; *, included in NMA

For quantitative analysis, analyzable data for NMA were available in only 19 studies for MACE outcome [10,11,14,27–30,32–34,38–40,42–44,46,48,49], eight for MI [11,14,15,27,32,34,48,49], five for stroke [11,15,27,34,38], but not enough data were available for CHF. Unfortunately, PAD was included in the MACE composite criteria in the studies but were not reported separately. Three studies [18,36,47] responded to our inclusion criteria but did not reported the number of MACE event, only Hazard Ratio (HR) or only the total number or cardiovascular events (not for each group) which have not been reported in Table 1.

### MACE risk with TCZ vs other bDMARD

The analysis of MACE outcome encompassed 19 studies (11 RCT and 8 cohorts) 12 312 patients were treated by TCZ, 8 123 with RTX, 28 728 with ABA, 45 963 with TNFi and 21 372 with csDMARD. 1 766 MACE were recorded: 125 under TCZ (1.02%), 183 under RTX (2.25%), 656 under ABA (2.28%), 1066 under TNFi (2.32%), 264 under csDMARD (1.24%).

**Fig 2:**
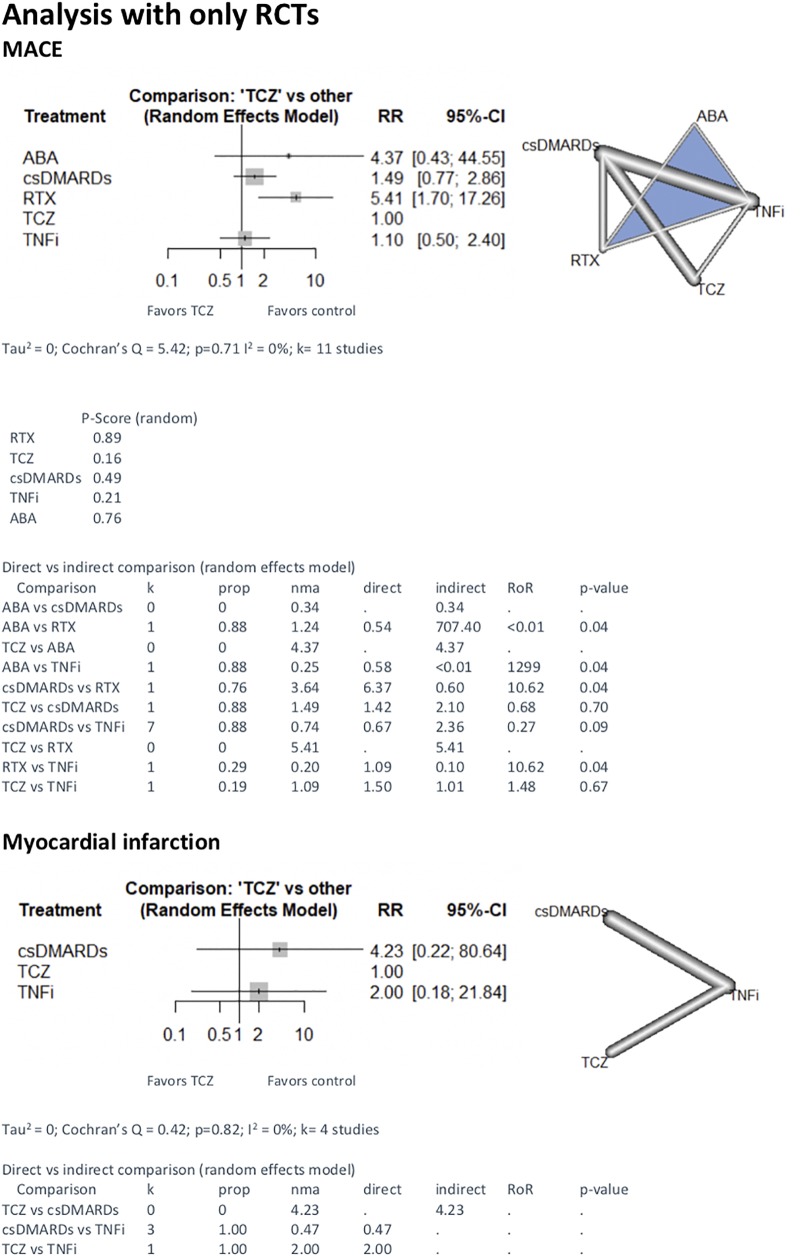
NMA with RCT on major adverse cardiac events, myocardial infarction and stroke with TCZ vs other treatments. comparison—Treatment comparison, k—Number of studies providing direct evidence, prop—Direct evidence proportion, nma—Estimated treatment effect (RR) in network meta-analysis, direct—Estimated treatment effect (RR) derived from direct evidence, indirect—Estimated treatment effect (RR) derived from indirect evidence, RoR—Ratio of Ratios (direct versus indirect), p-value—p-value of test for disagreement (direct versus indirect), TCZ—tocilizumab; csDMARDs—conventional synthetic disease-modifying antirheumatic drugs; TNFi—tumor necrosis factor inhibitor; ABA—abatacept; RTX—rituximab.

**Fig 3:**
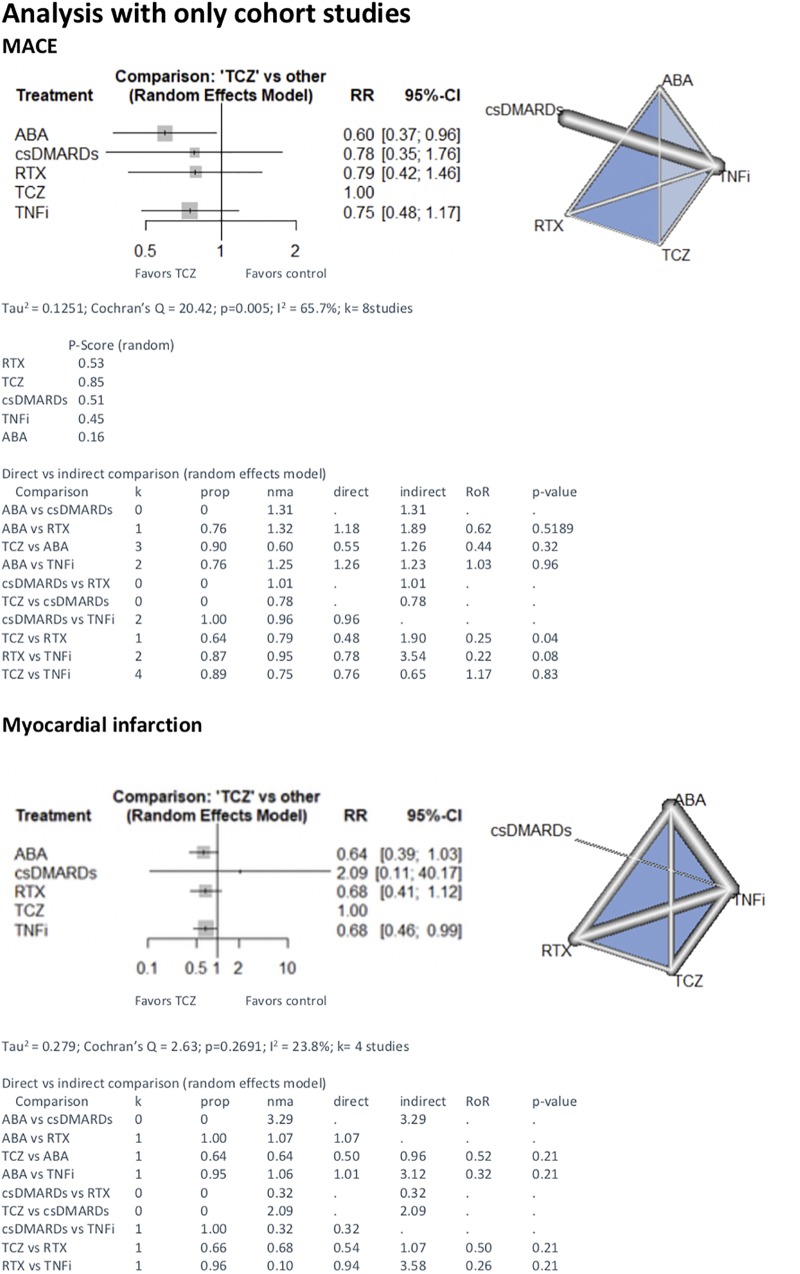
NMA with cohort studies on major adverse cardiac events, myocardial infarction and stroke with TCZ vs other treatments. comparison—Treatment comparison, k—Number of studies providing direct evidence, prop—Direct evidence proportion, nma—Estimated treatment effect (RR) in network meta-analysis, direct—Estimated treatment effect (RR) derived from direct evidence, indirect—Estimated treatment effect (RR) derived from indirect evidence, RoR—Ratio of Ratios (direct versus indirect), p-value—p-value of test for disagreement (direct versus indirect), TCZ—tocilizumab; csDMARDs—conventional synthetic disease-modifying antirheumatic drugs; TNFi—tumor necrosis factor inhibitor; ABA—abatacept; RTX—rituximab.

First, we included only RCT (n = 11) in the NMA. We found less events with RTX (TCZ vs RTX 5.41 [1.70;17.26]. We found no difference between TCZ and other treatments (1.10 [0.50;2.40] with TNFi, 4.37 [0.43;44.55] with ABA and 1.49[0.77;17.26] with csDMARD) (Fig 2). The results with only observational studies are represented in Fig 3.

**Fig 4:**
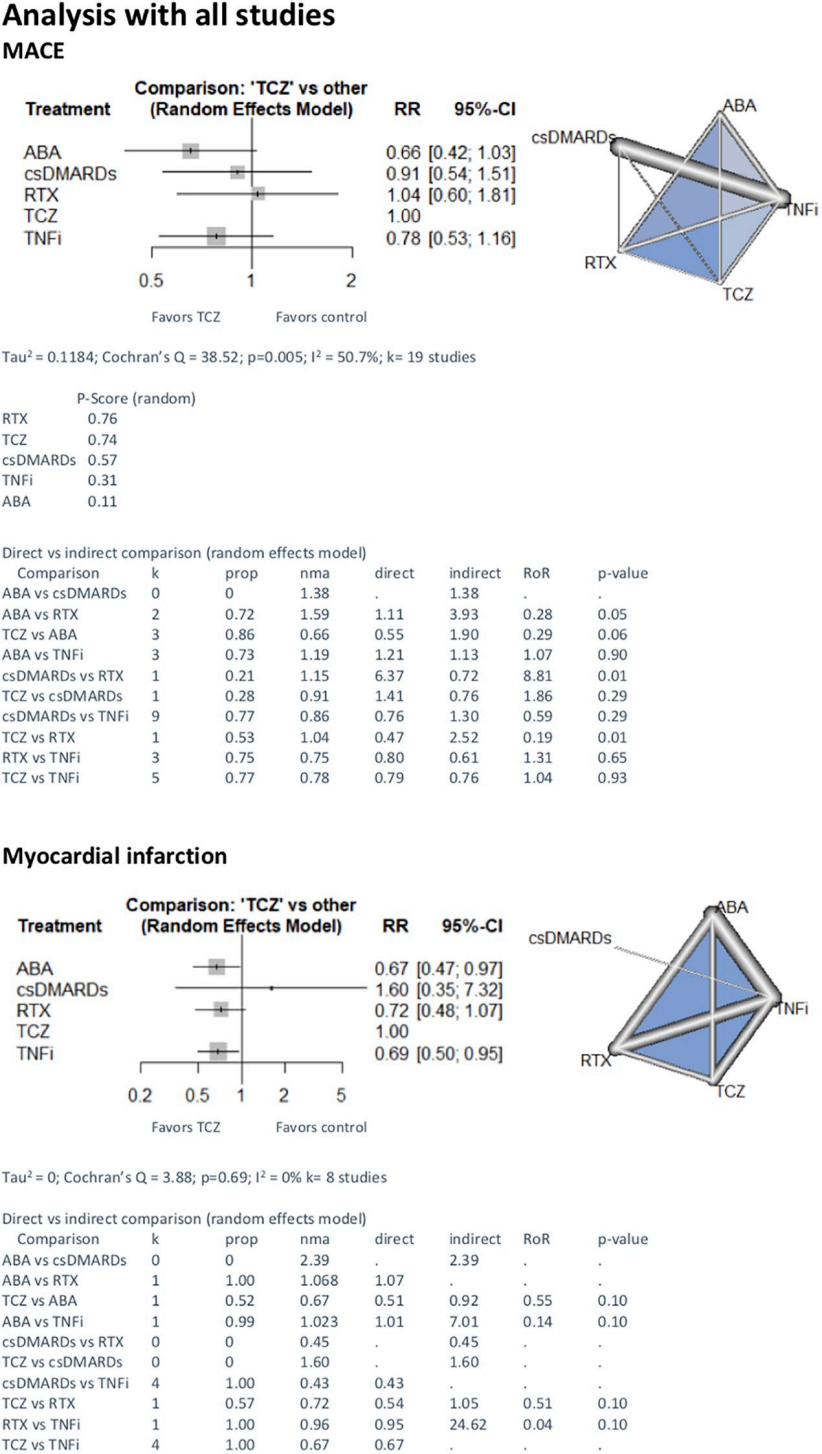
NMA with both designs (RCT and cohorts) on major adverse cardiac events, myocardial infarction and stroke with TCZ vs other treatments. comparison—Treatment comparison, k—Number of studies providing direct evidence, prop—Direct evidence proportion, nma—Estimated treatment effect (RR) in network meta-analysis, direct—Estimated treatment effect (RR) derived from direct evidence, indirect—Estimated treatment effect (RR) derived from indirect evidence, RoR—Ratio of Ratios (direct versus indirect), p-value—p-value of test for disagreement (direct versus indirect), TCZ—tocilizumab; csDMARDs—conventional synthetic disease-modifying antirheumatic drugs; TNFi—tumor necrosis factor inhibitor; ABA—abatacept; RTX—rituximab.

When we included both designs (RCT and cohort) we found no difference between TCZ and other treatments: 0.66 [0.42;1.03] with ABA, 1.04 [0.60;1.81] with RTX, 0.78[0.53;1.16] and 0.91 [0.54;1.51] with csDMARD (Fig 4).

### MI risk with TCZ vs other bDMARD

The analysis of MI risk included eight studies (four RCT and four cohorts). 11 723 patients were treated by TCZ, 74 75 with RTX, 13 608 with ABA, 53 068 with TNFi and 648 with csDMARD. 689 MI were recorded, 58 under TCZ (0.49%), 71 under RTX (0.95%), 138 under ABA (1.01%) and 422 under TNFi (0.80%) and no MI under csDMARD. In the RCT NMA we found no difference between TCZ and csDMARD (4.23 [0.22;80.64]), no difference between TCZ and TNFi (2.00 [0.18;21.84]). We lacked data to compare TCZ and other bDMARD (Fig 2). With both designs included, the risk of myocardial infarction was lower with TCZ compared to ABA (0.67 [0.47;0.97]). There was no difference with other treatments (Fig 4).

### Stroke risk with TCZ vs other bDMARD

Five studies were included for stroke event (two RCT and three cohorts). 11 345 patients were treated by TCZ, 22 024 by TNFi and 363 by csDMARD. 119 strokes were recorded, 40 under TCZ (0.35%), 79 under TNFi (0.36%). We did not record stroke event under csDMARD only. With RCT NMA, there was no difference between TCZ and csDMARD (1.51[0.02;103.50] and between TCZ and TNFi (1.00 [0.06;15.85]). We lacked data to compare TCZ and other bDMARD (Fig 2). Similar results were found with the analysis that included both designs (Fig 4), and with observational studies only (Fig 3).

### Comparison of CHF

There was not enough data available to perform a network meta-analysis.

## Discussion

We performed the first network meta-analysis comparing the cardiovascular outcomes of TCZ and other biologics. However, we found a lower risk of MACE with RTX in the RCT NMA, which is not significant when we included only cohorts or both designs. This study showed that TCZ has similar cardiovascular outcomes comparing with other bDMARD and csDMARD.

TCZ significantly increases lipids levels more than TNFi [12], ABA and RTX [50]. This is one of the reasons why TCZ was initially used with a particular attention in patient with a high cardiovascular risk. Despite this increase, our results show the cardiovascular safety profile of TCZ comparing to the other biologics, especially when we included only RCT in the NMA. We also found less myocardial infarction with TCZ than ABA and TNFi in observational studies. This beneficial effect on myocardial infarction could be explained by the fact that TCZ decreases more lipoprotein A levels, and SAA-HDL than adalimumab in the ADACTA study [12]. Indeed, lipoprotein-A has been associated with MI [51] and the HDL linkage by SSA protein could be responsible for HDL-C loss anti atherogenic function [12,50,52,53]. Furthermore, inflammatory cytokines, particularly IL-1 and IL-6 seem to play a determinant role in the atherosclerotic process. Recently, the CANTOS trial performed in general population shown a significantly lower rate of recurrent cardiovascular events with canakinumab than placebo [54]. A cohort study found an improvement of the endothelial function in high risk patients with RA with TCZ but not with TNFi [55]. IL-6 seems strongly involved in the coronary heart disease (CHD), especially via the trans-signaling pathway [56–58]. Mendelian randomization studies showed that IL-6 up-regulation increases CHD risk [59,60]. Conversely, the variant Asp358 causes an increase in the soluble IL-6 receptor (IL-6Rs) and is associated with a reduced risk of coronary artery disease [61,62]. Indeed, in a placebo-RCT in general population with MI, a single dose of TCZ decreases more troponin levels than placebo at day 3 [63]. In an observational study Kobayashi & al. found that TCZ treatment in RA patients significantly increased left ventricular ejection fraction and decreased left ventricular mass index comparing to healthy controls [64]. Finally, in high-risk population of CHD, high IL-6 levels were associated with MI risk, cardiovascular death and all causes death [65].

This systematic review and meta-analysis have several strengths. Indeed, we performed an exhaustive review, through four international databases, and two international meetings databases. The included studies showed high quality. RCT were at low risk of bias except for which who were open-label. However, one of these had MACE as primary outcome with an open-label design. Furthermore this study is not published and was found in the 2016 ACR meetings abstract [16]. Most of the cohort studies were at “moderate risk of bias” only because of their non-randomized design. We realized a network meta-analysis which allowed us to make indirect comparisons of multiple treatments and thus to analyze a large amount of studies including placebo RCT. Due to multiple comparators, heterogeneity had to be measured which was possible with this technique. Furthermore, we used random effects model even if the heterogeneity test was no statistically significant because it considers fluctuations of sampling and also treatment effect fluctuations due to multiple covariates. Finally, although we restricted our research to English and French languages, no article was excluded because of the language.

However, our studies showed some limits. The heterogeneity test was significant for MACE outcome which was controlled by using a random effects model. The transivity hypothesis has not been tested, which is a limitation of the study. Unfortunately, the baseline CV risk was not specified in the included studies, we were unable to carry out an analysis to determine whether this risk was an effect modifier.

Our network meta-analysis illustrates the indication bias in observational studies. Indeed, the beneficial effect of TCZ for MI could be explained by a lesser use of TCZ in high CV risk patients because of the cholesterol increase under treatment. The fact that TCZ is under used in high-risk CV patients is not supported by the literature, the indication bias due to the prudent use of TCZ in high-risk patients is therefore purely hypothetical, not supported by the literature, but remains likely. Another explanation is that observational studies were designed to study cardiovascular events, with high sample sizes, unlike RCTs.

## Conclusion

Despite these limits, our study showed a good cardiovascular safety profile of TCZ compared to other bDMARD, with potential benefit on MI compared to other bDMARD. Further studies are needed to corroborate these data.

## Supporting information

S1 File PRISMA checklist. (DOC)

S2 File Research equations. Medline, Cochrane library, Web of science, Science Direct, EULAR and ACR databases.
(DOCX)
