# Supplementary material for: Cardiovascular safety of tocilizumab: A systematic review and network meta-analysis
Source: PLoS One. 2019 Aug 1;14(8):e0220178. doi: 10.1371/journal.pone.0220178 (PMC6675055; doi:10.1371/journal.pone.0220178)
Supplement: S2 File — Medline, Cochrane library, Web of science, Science Direct, EULAR and ACR databases. (DOCX) [file pone.0220178.s002.docx]

**S1 File. Research equations.**

# Our research equations were deliberately quite broad and we wanted to obtain all studies concerning biotherapies in RA, including efficacy studies to look for cardiovascular events in safety outcomes

# Medline via Pubmed

# First, we used MESH terms from January 2003 until May 2018.

# We choose 2003 because it was the beginning of the commercialization of biologic treatments such as TNF inhibitors.

(((((((("tocilizumab" [Supplementary Concept]) OR "golimumab" [Supplementary Concept]) OR "Certolizumab Pegol"[Mesh]) OR "Etanercept"[Mesh]) OR "Infliximab"[Mesh]) OR "Adalimumab"[Mesh]) OR "Rituximab"[Mesh]) OR "Abatacept"[Mesh]) AND "Arthritis, Rheumatoid"[Mesh]

Then we used the same search equation in free text in order to detect publication not MESH referenced.

((((((((tocilizumab) OR golimumab) OR Certolizumab Pegol) OR Etanercept) OR Infliximab) OR Adalimumab) OR Rituximab) OR Abatacept) AND Rheumatoid Arthritis

N=8524, filtre 2017-2018

# Cochrane Library

tocilizumab AND cardiovascular (title, Abstract, Keyword)

# Science direct / Elsevier database

+Rheumatoid arthritis +(abatacept OR infliximab OR golimumab OR TNF inhibitors OR certolizumab OR etanercept OR adalimumab OR tocilizumab OR rituximab) +(safety OR efficacy OR cardiovascular)

# Web of Science

TOPIC:(rheumatoid arthritis) AND TOPIC:(tocilizumab OR abatacept OR rituximab OR TNF inhibitor OR anti TNF OR golimumab OR infliximab OR adalimumab OR etanercept OR certolizumab) AND TITLE: (safety OR efficacy OR cardiovascular)

Timespan: All years. Indexes: SCI-EXPANDED, SSCI, A&HCI, CPCI-S, CPCI-SSH, ESCI, CCR- EXPANDED, IC.

# Abstracts ACR from 2012 to 2017

# We choose 2012 because there was no abstract available before this date. We can’t include 2018 abstract because the congress had not yet taken place.

((((((((tocilizumab) OR golimumab) OR Certolizumab Pegol) OR Etanercept) OR Infliximab) OR Adalimumab) OR Rituximab) OR Abatacept) AND Rheumatoid Arthritis

**EULAR abstract from 2014 to 2017**

# We choose 2014 because there was no abstract available before this date. We can’t include 2018 abstract because the congress had not yet taken place.

((((((((tocilizumab) OR golimumab) OR Certolizumab Pegol) OR Etanercept) OR Infliximab) OR Adalimumab) OR Rituximab) OR Abatacept) AND Rheumatoid Arthritis
